# Targeting chemotherapy-induced PTX3 in tumor stroma to prevent the progression of drug-resistant cancers

**DOI:** 10.18632/oncotarget.4364

**Published:** 2015-06-08

**Authors:** Jhih-Ying Chi, Yu-Wei Hsiao, Chien-Feng Li, Yu-Chih Lo, Zu-Yau Lin, Jhen-Yi Hong, Yang-Ming Liu, Xiu Han, Shao-Ming Wang, Ben-Kuen Chen, Kelvin K. Tsai, Ju-Ming Wang

**Affiliations:** ^1^ Institute of Basic Medical Science, National Cheng Kung University, Tainan, Taiwan R.O.C; ^2^ Institute of Bioinformatics and Biosignal Transduction, National Cheng Kung University, Tainan, Taiwan R.O.C; ^3^ Department of Pathology, Chi-Mei Medical Center, Tainan, Taiwan R.O.C; ^4^ Cancer Center and Division of Hepatobiliary Medicine, Department of Internal Medicine, Kaohsiung Medical University Hospital, Taiwan R.O.C; ^5^ National Institute of Cancer Research and Translational Center for Glandular Malignancies, National Health Research Institutes, Tainan, Taiwan R.O.C; ^6^ Institute of Medical Sciences, Taipei Medical University, Taipei, Taiwan R.O.C

**Keywords:** CEBPD, PTX3, TAMs, CAFs, tumor microenvironment

## Abstract

The tumor microenvironment has been suggested to participate in tumorigenesis, but the nature of the communication between cancer cells and the microenvironment, especially in response to anticancer drugs, remains obscure. We determined that activation of the CCAAT/enhancer binding protein delta (CEBPD) response to Cisplatin and 5-Fluorouracil in cancer-associated macrophages and fibroblasts contributed to the metastasis, invasion, acquired chemoresistance and stemness of cancer cells by *in vitro* and *in vivo* assays. Specifically, reporter and *in vivo* DNA binding assays were used to determine that *Pentraxin 3* (*PTX3*) is a CEBPD responsive gene and serves a protumor role upon anticancer drug treatment. Finally, a PTX3 peptide inhibitor RI37 was developed and assessed the antitumor effects by *in vivo* assays. RI37 could function as a promising inhibitor for preventing cancer progression and the metastasis, invasion and progression of drug-resistant cancers. The identification of PTX3 provided a new insight in the interaction between host and tumor and the RI37 peptide showed a great opportunity to largely reduce the risk of invasion and metastasis of cancer and drug-resistant cancers.

## INTRODUCTION

Anticancer drug resistance is one of the major challenges in cancer therapy. The majority of cancer patients receive chemotherapy after surgical intervention. However, even when chemotherapy appears successful, cancer patients still face the risk of recurrence of the same or a drug-resistant cancer. Therefore, understanding the relationship between host and tumor may inform the identification and design of more effective therapies to overcome the spread or recurrence of cancer and improve the outcome of cancer therapies.

Previous studies have indicated that the tumor microenvironment provides a protective niche for cancer progression [1-4]. Clinical evidence also showed a strong association between the number of tumor-associated macrophages (TAMs) and cancer-associated fibroblasts (CAFs) and poor prognosis in several types of cancer [5-8]. TAMs are primarily a macrophage subpopulation with an M2 phenotype [9] and the CAFs detected are predominantly myofibroblasts [10], both of which respond differently to various microenvironmental signals and serve as a source of cytokines released in the cancer-initiating cell microenvironment [11]. However, the responses of M2 macrophages/TAMs and myofibroblasts/CAFs to anticancer drugs and the consequent effects, including the metastasis, invasion, acquired chemoresistance and stemness of cancer cells, remain largely uninvestigated.

CEBPD belongs to the CCAAT/enhancer binding protein (C/EBP) family of transcription factors. Under normal physiological conditions, CEBPD is not highly expressed, but it can be upregulated by a variety of extracellular stimuli, including IL-1β, prostaglandin E2 (PGE2) and TNFα [12-14]. CEBPD regulates or co-regulates a wide range of inflammatory factors, such as TNFα, IL-1β, IL-6, CXCL1 [15], chemokines, monocyte chemoattractant protein 1 (MCP1) and IL-10 [16, 17]. CEBPD is thought to function as a tumor suppressor in cancer cells because the loss of CEBPD promotes tumor progression [18]. Clinically, the silencing of CEBPD has been observed in many cancers, including cervical cancer, hepatocellular carcinoma, prostate cancer, leukemia and breast cancer [15]. We previously demonstrated that the polycomb group complex and DNA methyltransferase mediated the hypermethylation of the CEBPD promoter in cervical cancer cells [18]. Interestingly, a recent study showed an increase in mammary tumor multiplicity and a decrease in lung metastasis in *Cebpd^−/−^/HER2/neu* mice [19]. This result is consistent with the possibility that CEBPD acts as a tumor suppressor in cancer cells but may contribute to invasion and metastasis in stromal cells in the tumor microenvironment. Importantly, our study showed that an increase in the CEBPD level in M2 macrophages in response to PGE2 resulted in enhanced production of IL-10 and PTX3 and had a protumor effect [17]. However, the details of CEBPD biology in the tumor microenvironment, as well as its biological impacts and potential application in cancer therapy, remain largely unknown.

Pentraxins are a family of evolutionarily conserved proteins that function at the crossroads between innate immunity, inflammation, matrix deposition and female fertility [20]. PTX3 consists of a C-terminal domain similar to those of classical pentraxins, such as C-reactive protein and serum amyloid P component, and an unrelated N-terminal domain [21]. PTX3 synthesis is stimulated by a variety of molecules that participate in the inflammatory process, including LPS, IL-1β and TNFα [22, 23]. PTX3 is mainly composed of octamers that are covalently linked by intra- and inter-chain disulfide bonds [24] and glycosylation has been suggested to modulate PTX3 function during inflammation [23]. Previously, we found that PTX3 is a downstream target of CEBPD in macrophages and functions to reduce the macrophage-mediated phagocytosis of nasopharyngeal carcinoma cells. Moreover, although a high PTX3 abundance has been observed in the serum of several cancers, including liposarcoma, prostate cancer, lung cancer and breast cancer [25-27], the precise role of PTX3 in tumorigenesis remains largely uninvestigated.

In this study, we elucidated the biology of the CEBPD response to anticancer drugs in cancer-associated macrophages and fibroblasts. We found that activation of CEBPD in the tumor microenvironment contributed to the metastasis, invasion, acquired chemoresistance and stemness of cancer cells. We next demonstrated that PTX3, a gene that is directly regulated by CEBPD, can fully support the protumor role of CEBPD in the tumor microenvironment. We further developed a PTX3 inhibitor RI37 peptide to prevent CEBPD/PTX3 axis-induced cancer malignancies and reduce the metastasis and invasion of drug-resistant cancers.

## RESULTS

### Activation of CEBPD in M2 macrophages and myofibroblasts/CAFs contributes to the acquired chemoresistance, stemness, migration and invasion of cancer cells

**Figure 1:**
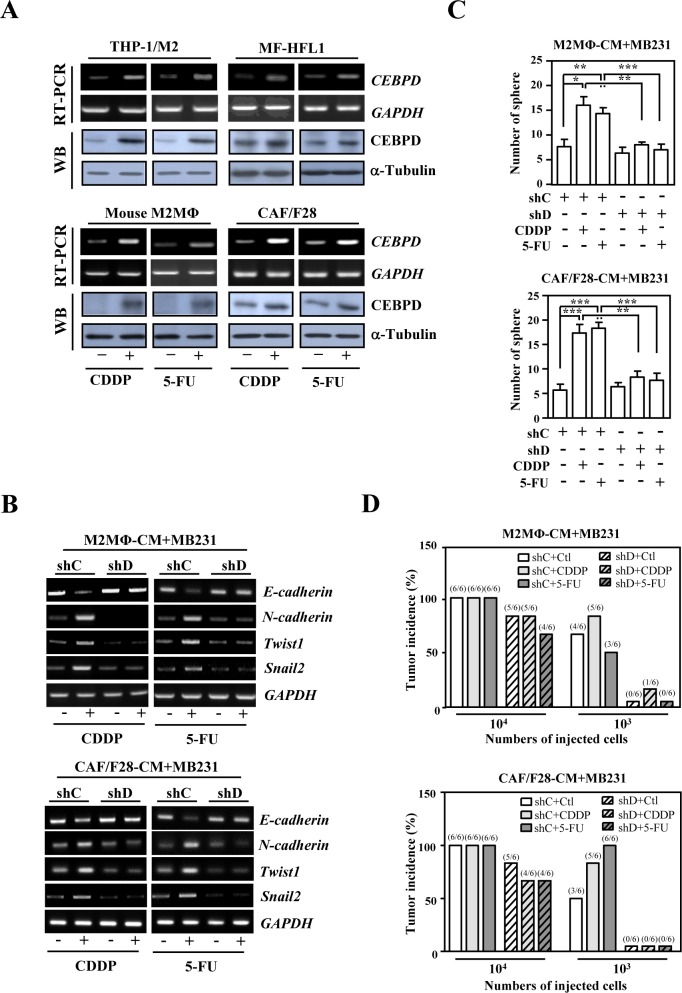
The activation of CEBPD in M2-like macrophages and myofibroblasts/CAFs enhances the sphere formation of breast cancer cells upon anticancer drug treatment **A.** *CEBPD* transcript and protein levels were increased upon CDDP and 5-FU treatment in THP-1/M2 cells, mouse M2-like macrophages, HFL1 myofibroblasts and CAF/F28 cells. **B.** CEBPD-expressing THP-1/M2 or CAF/F28 cells regulated transcription of EMT (*E-cadherin* and *N-cadherin*) and cancer stem cell (*Twist1* and *Snail2*) markers in MB231 cells. An RT-PCR assay was conducted using total RNA harvested from MB231 cells cultured with conditioned medium from THP-1/M2 or CAF/F28 cells infected with shC or shD lentiviruses and treated with or without CDDP or 5-FU. **C.** The loss of CEBPD in THP-1/M2 (M2MΦ) or CAF/F28 cells attenuated sphere formation by MB231 cells. MB231 cells were grown in conditioned medium from THP-1/M2 or CAF/F28 cells infected with lentiviruses bearing shLacZ (shC) or shCEBPD (shD) and treated with or without CDDP or 5-FU. **D.** MB231 cells (10^3^ or 10^4^) collected from spheres in Figure 1C were subcutaneously inoculated into NOD-SCID mice. The incidence of MB231 xenografted tumors was calculated after 30 days.

The critical role of the microenvironment in cancer progression and response to therapies, such as radiation and anticancer drugs, is being increasingly recognized [1, 2, 28-31]. However, the molecular components of the interaction between cancer and host cells remain largely uninvestigated. Importantly, as well as breast cancer cells (Fig. S1), the transcription factor CEBPD was induced in THP-1 M2-like macrophages (THP-1/M2), mouse M2 macrophages, HFL1 myofibroblasts (HFL1-MF) cells and cancer associated fibroblast/F28 (CAF/F28) cells upon treatment with Cisplatin (CDDP) or 5-Fluorouracil (5-FU) (Figure. 1A). This observation prompted us to examine whether anticancer drug-induced upregulation of CEBPD in the tumor microenvironment contributed to the stemness of cancer cells and to the metastasis and invasion of anticancer drug-resistant cancer cells.

First, we found that conditioned media from CEBPD-expressing THP-1/M2 or CAF/F28 cells attenuated *E-cadherin* and increased *N-cadherin*, *Twist1* and *Snail2* transcripts in MDA-MB231 (MB231) cells (Figure. 1B) and enhanced sphere formation of MB231 cells (Figure. 1C). Moreover, conditioned media from CDDP- or 5-FU-treated THP-1/M2 or CAF/F28 cells lacking CEBPD reverse the expression of EMT/stemness markers and significantly inhibited this sphere-forming ability *in vitro* (Figure. 1B and 1C) and tumorigenicity in NOD/SCID mice (Figure. 1D). These results indicate that increased expression of CEBPD in M2 macrophages and myofibroblasts/CAFs contributes to the stemness of cancer cells. We also assessed whether CEBPD in THP-1/M2 cells and CAF/F28 cells contributed to the anticancer drug resistance exhibited by cancer cells. We found that MB231 cells were sensitized to CDDP and 5-FU when they were cultured in conditioned media from THP-1/M2 or CAF/F28 cells lacking CEBPD (Figure. S2).

**Figure 2:**
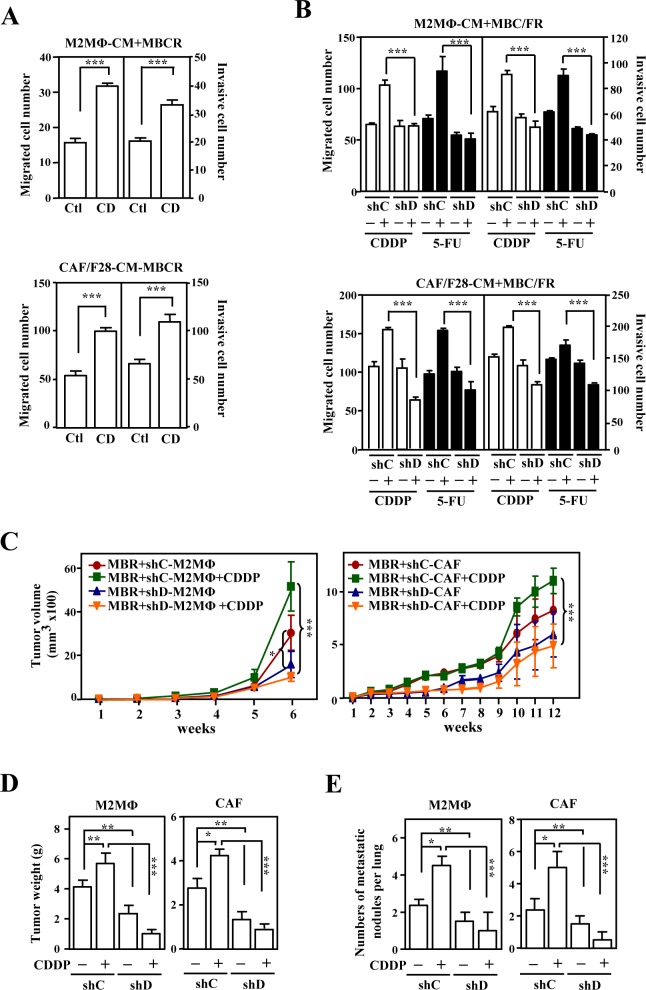
The activation of CEBPD after anticancer drug treatment in M2-like macrophages and CAFs enhances growth and metastasis/invasion of cancer cells **A.** The migration and invasion of mcCDRMB231 (MBCR) cells were assessed using a Boyden chamber assay in which MBCR cells were cultured in conditioned medium from THP-1/M2 (M2MΦ) or CAF/F28 cells infected with lentiviruses bearing an empty vector (Ctl) or CEBPD expression vector (CD). **B.** The migration and invasion of mcCDRMB231 (MBCR) and mcFURMB231 (MBFR) cells were assessed using a Boyden chamber assay in which cells were cultured in conditioned medium from THP-1/M2 (M2MΦ) or CAF/F28 cells infected with lentiviruses with shLacZ (shC) or shCEBPD (shD) and treated with or without CDDP or 5-FU. **C.** mcCDRMB231 (MBR) cells were implanted into immunodeficient NOD-SCID mice. Stable THP-1/M2 cells bearing shC (shC-M2MΦ) or shD (shD-M2MΦ) vectors or CAF/F28 cells bearing shC (shC-CAF) or shD (shD-CAF) vectors were subcutaneously co-inoculated with mcCDRMB231 (MBR) cells into NOD-SCID mice. The mice were treated with or without CDDP (5 mg/kg). Tumor size was measured with external calipers and tumor volume was calculated using the standard formula: *V* = (*w* x *l*^2^) x 0.52, where *l* is the length and *w* is the width of the tumor (n=6 per group). The mice with mcCDRMB231-xenografted tumors in Figure 2C were sacrificed in the 6^th^ or 12^th^ week. Tumor weight was measured as described above and the results are shown in **D.**. The metastasis of mcCDRMB231-xenografted tumors to the nodules of lung tissues was determined and a statistic result is shown in **E.**.

Next, following the generation of CDDP-resistant mCherry fluorescent MB231 (mcCDRMB231), 5-FU-resistant mCherry fluorescent MB231 (mcFURMB231), CDDP-resistant mCherry fluorescent 4T1 (mcCDR4T1) and 5-FU-resistant mCherry fluorescent 4T1 (mcFUR4T1) cell lines (Figure. S3), we assessed the effect of anticancer drug-induced CEBPD in M2 macrophages and CAFs on the metastasis and invasion of drug-resistant cancer cells. Conditioned medium from CEBPD-expressing THP-1/M2 or CAF/F28 cells promoted the migration and invasion of mcCDRMB231 cells (Figure. 2A). Moreover, conditioned media from CDDP- or 5-FU-stimulated THP-1/M2 cells, mouse M2 macrophages, HFL1-MF cells and CAF/F28 cells also promoted the migration and invasion of mcCDRMB231, mcFURMB231, mcCDR4T1 and mcFUR4T1 cells (Figure. 2B and Figure. S4A). In contrast, these effects were attenuated when conditioned medium from M2 macrophages or myofibroblasts/CAFs lacking CEBPD was used (Figure. 2B and Figure. S4A).

We further assessed the *in vivo* effects of activated CEBPD in stroma on the CDDP treatment of CDDP-resistant cancer cells. First, mcCDRMB231 cells were co-transplanted with THP-1/M2 cells carried control knockdown vector (shC-M2MΦ) or CEBPD knockdown vector (shD-M2MΦ) or CAF/F28 cells with control knockdown vector (shC-CAF) or CEBPD knockdown vector (shD-CAF) into NOD-SCID mice. After CDDP treatment, the mcCDRMB231 cells co-transplanted with shC-M2MΦ cells showed greater growth than those co-transplanted with shD-M2MΦ (Figure. 2C and 2D). Moreover, cancer growth was enhanced in mcCDRMB231/shC-CAF co-transplants upon CDDP treatment compared with mcCDRMB231/shD-CAF co-transplants (Figure. 2C and 2D). In agreement with the results showing increased growth compared with THP-1/M2 or CAF/F28 cells without CEBPD, the number of metastatic and invasive mcCDRMB231 cells in the lungs was increased in co-transplants with shC-M2MΦ or shC-CAF cells upon CDDP treatment (Figure. 2E). Next, mcCDR4T1 cells were co-transplanted with mouse *Cebpd*^+/+^ or *Cebpd*^−/−^ M2 macrophages or myofibroblasts into NOD-SCID mice. After CDDP treatment, mcCDR4T1 tumors were larger in size and showed greater metastatic activity when co-transplanted with *Cebpd*^+/+^ M2 macrophages or myofibroblasts than co-transplants with *Cebpd*^−/−^ M2 macrophages or myofibroblasts (Figure. S4B). These results suggest that drug-induced CEBPD activation in the tumor microenvironment promotes the migration and invasion of chemoresistant cancers.

### CEBPD upregulates PTX3 in M2 macrophages and myofibroblasts/CAFs

**Figure 3:**
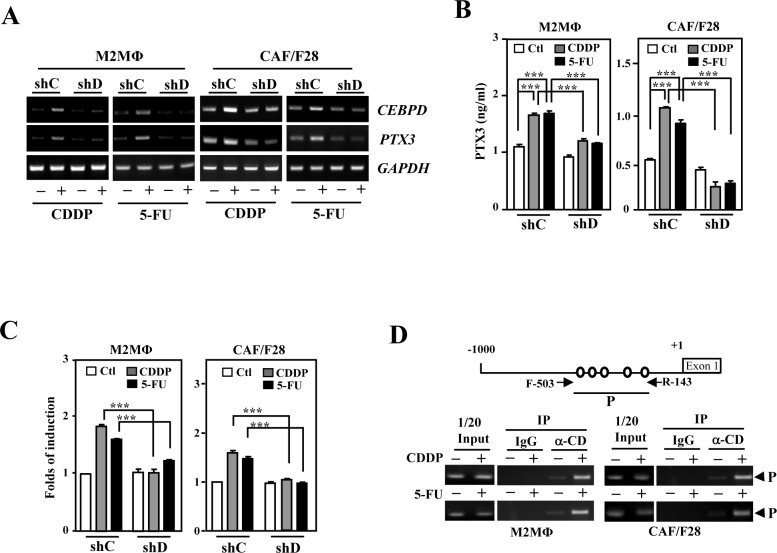
PTX3 is activated upon induction of CEBPD in M2-like macrophages and myofibroblasts/CAFs **A.** The levels of PTX3 induced following CEBPD induction by CDDP or 5-FU treatment in THP-1/M2 (M2MΦ) and CAF/F28 cells. THP-1/M2 (M2MΦ) or CAF/F28 cells were infected with shLacZ (shC) and shCEBPD (shD) lentiviruses. **B.** Following preincubation with shC or shD lentiviruses, an ELISA was performed to detect PTX3 secretion in CDDP- or 5-FU-treated THP-1/M2 (M2MΦ) or CAF/F28 cells. **C.** Following preincubation with shC or shD lentiviruses, a reporter assay was performed using the lysates of CDDP- or 5-FU-treated THP-1/M2 (M2MΦ) or CAF/F28 cells transfected with the *PTX3* reporter. **D.** A PCR assay was performed using the purified fragmented gDNA of CDDP- or 5-FU-treated THP-1/M2 (M2MΦ) and CAF/F28 cells and the indicated antibody-immunoprecipitated products following the ChIP assay.

We previously demonstrated that CEBPD-induced PTX3 in macrophages promotes cancer progression by preventing the macrophage-mediated phagocytosis of tumor cells [17]. To further dissect the participation and regulation of the *PTX3* gene in response to CDDP and 5-FU in the tumor microenvironment, the expression of *PTX3* gene and the effects of the PTX3 were investigated, respectively, by following *in vitro* and *in vivo* assays. The *PTX3* transcript and protein levels were significantly increased in response to both CDDP and 5-FU in THP-1/M2 and CAF/F28 cells (Figure. 3A and 3B). The CDDP- and 5-FU-induced *PTX3* reporter activities were attenuated by the knockdown of CEBPD (Figure. 3C). An *in vivo* DNA binding assay further showed that the binding of CEBPD to the *PTX3* promoter was induced upon CDDP or 5-FU treatment (Figure. 3D). These results indicate that CEBPD activates *PTX3* transcription in response to treatment with CDDP or 5-FU by directly binding to its promoter region in M2 macrophages and CAFs.

### PTX3 is involved in the CEBPD-induced acquired chemoresistance, stemness and metastasis/invasion of cancer cells

**Figure 4:**
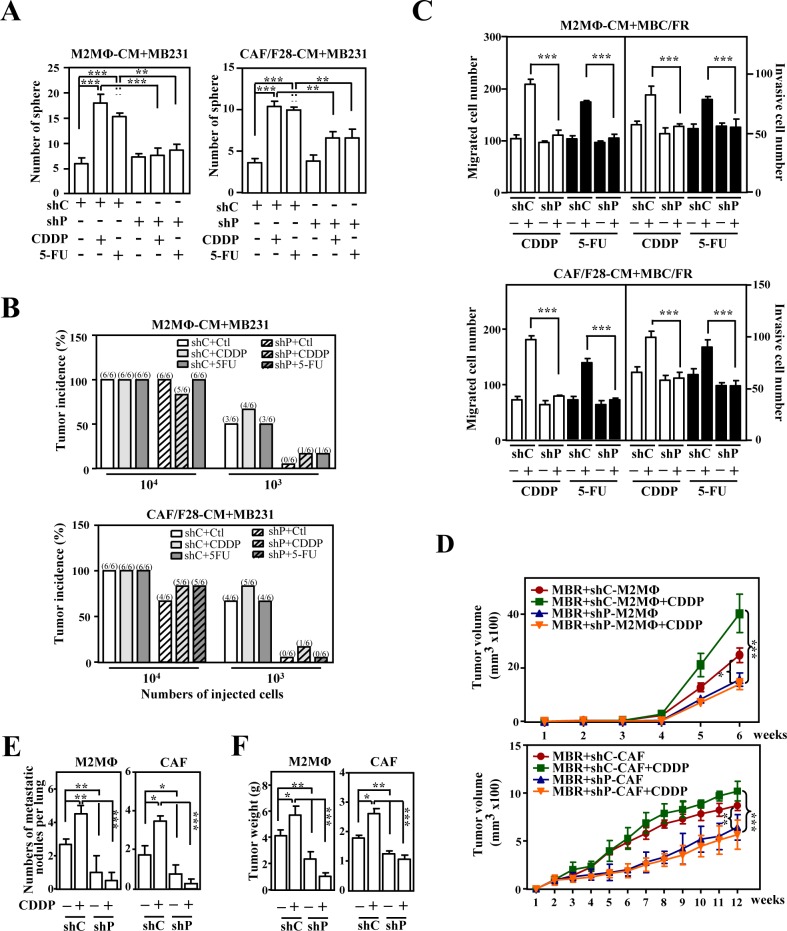
PTX3 contributes to the stemness, metastasis and invasion of cancer cells **A.** The loss of PTX3 in THP-1/M2 (M2MΦ) or CAF/F28 cells attenuated sphere formation by MB231 cells. MB231 cells were grown in conditioned medium from THP-1/M2 or CAF/F28 cells infected with lentiviruses bearing shLacZ (shC) or shPTX3 (shP) and treated with or without CDDP or 5-FU. **B.** MB231 cells (10^3^ or 10^4^) collected from the spheres in Figure 4A were subcutaneously inoculated into NOD-SCID mice. The incidence of MB231-xenografted tumors was calculated after 30 days. **C.** The migration and invasion of mcCDRMB231 (MBCR) and mcFURMB231 (MBFR) cells were assessed using a Boyden chamber assay in which the cells were cultured in conditioned medium from THP-1/M2 (M2MΦ) or CAF/F28 cells infected with shC or shP lentiviruses with or without CDDP or 5-FU treatment. **D.** mcCDRMB231 (MBR) cells were implanted into immunodeficient NOD-SCID mice. Stable THP-1/M2 cells bearing shC (shC-M2MΦ) or shP (shP-M2MΦ) vectors or CAF/F28 cells bearing shC (shC-CAF) or shP (shP-CAF) vectors were subcutaneously co-inoculated with mcCDRMB231 cells into NOD-SCID mice and the mice were treated with or without CDDP (5 mg/kg). Tumor size was measured as described above (*n* = 6 per group). The mice with MBR-xenografted tumors in Figure 4D were sacrificed in the 6^th^ or 12^th^ week. The metastasis of mcCDRMB231-xenografted tumors to lung tissue nodules was calculated and shown in **E.**. Tumor weight was measured as described above and the results are shown in **F.**.

We found that the activation of CEBPD in M2-like macrophages and CAFs contributed to stemness and the migration and invasion of cancer cells that were both susceptible and resistant to drugs. We further tested whether PTX3 contributed to CEBPD-enhanced protumor activity. Conditioned media from THP-1/M2 or CAF/F28 cells lacking PTX3 could reverse the *E-cadherin* transcripts but attenuated *N-cadherin, Twist1* and *Snail2* transcripts in MB231 cells (Figure. S5A). Moreover, the conditioned media from THP-1/M2 or CAF/F28 cells lacking PTX3 could decrease *in vitro* sphere-forming ability and *in vivo* tumorigenicity in MB231 xenografted NOD/SCID mice (Figure. 4A and 4B). It also increased sensitivity of MB231 cells in response to CDDP and 5-FU (Figure. S5B) and decreased the *in vitro* migration and invasion of CDDP-treated mcCDRMB231 or 5-FU-treated mcFURMB231 cells (Figure. 4C). We further assessed the effects of PTX3 by examining *in vivo* tumor growth and metastasis/invasion of mcCDRMB231 cells upon CDDP treatment in a PTX3-enriched or depleted environment. mcCDRMB231 cells were co-transplanted with THP-1/M2 or CAF/F28 cells into NOD-SCID mice. Upon CDDP treatment, the co-transplants that were stably expressing the PTX3 knockdown vector (shP) were smaller and exhibited less metastatic activity compared with co-transplants of mcCDRMB231 cells with shC-M2MΦ or shC-CAF cells (Figure. 4D-4F). These results indicate that activated PTX3 in M2 macrophages or CAFs promotes the growth, metastasis and invasion of drug-resistant cancers. The data also suggest that PTX3 could be a therapeutic target for the development of a potent anticancer drug.

### PTX3 may be a therapeutic target for preventing the stemness, migration and invasion of cancer cells

**Figure 5:**
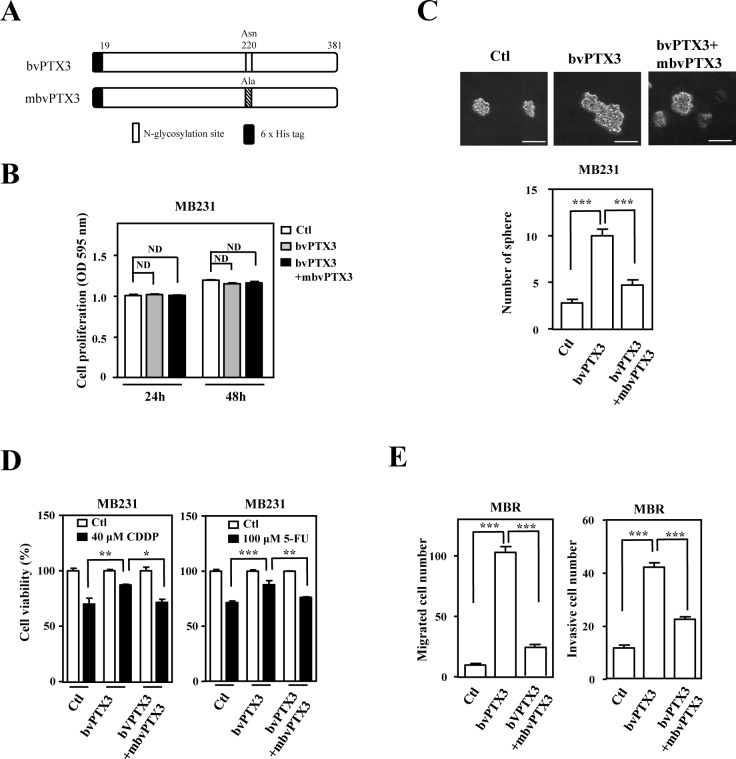
Mutant bvPTX3 (mbvPTX3) attenuates bvPTX3-induced stemness, drug resistance and migration/invasion of breast cancer cells **A**. A schematic representation of various recombinant forms of PTX3 (bvPTX3 or mbvPTX3). **B**. bvPTX3 and mbvPTX3 had no effect on the proliferation of MB231 cells. MB231 cells were exposed to the recombinant proteins mbvPTX3and mbvPTX3 and the proliferation of experimental cells was assessed at the 24th and 48th h. **C**. mbvPTX3 inhibited mbvPTX3-induced sphere formation by MB231 cells. MB231 cells were treated with mbvPTX3 or the Asn220-mutated mbvPTX3 [N-glycosylation site]. The number of spheres was calculated after 14 days. **D**. mbvPTX3 attenuated the mbvPTX3-induced drug resistance of MB231 cells. The viability of CDDP- or 5-FU-treated MB231 cells was calculated by incubating cells with mbvPTX3 or mbvPTX3 as indicated and further cell viability assays were conducted after 48 h. **E**. mbvPTX3 inhibited the mbvPTX3-induced *in vitro* migration and invasion of cancer cells. mcCDRMB231 (MBR) cells were seeded in the upper layer of a Boyden chamber. After 3 h, the culture medium was replaced with serum-free medium in the upper layer and bvPTX3 or mbvPTX3, as indicated, were added to serum-free medium in the bottom layer.

**Figure 6:**
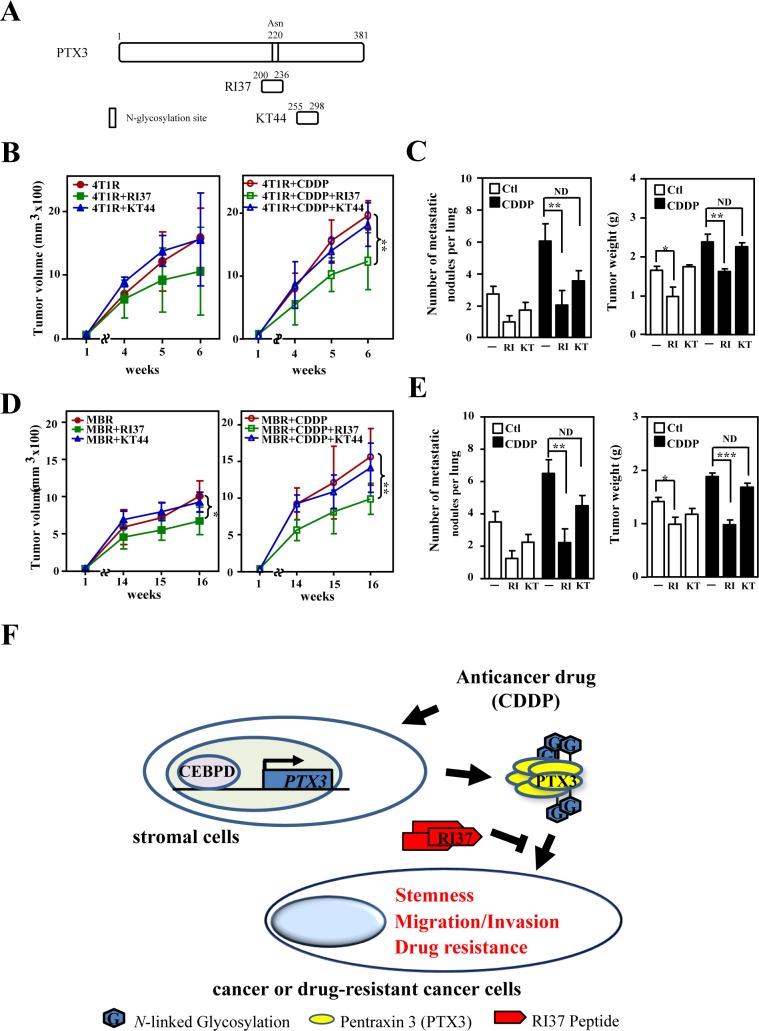
Peptide RI37, but not KT44, attenuates growth and metastasis of breast cancer cells **A.** A schematic representation of various PTX3 peptides (RI37 and KT44). **B.** mcCDR4T1 (4T1R) cells were subcutaneously inoculated into wild-type BALB/c mice. One week later, CDDP with or without RI37 or KT44 were administered to the experimental mice as indicated. Tumor size was measured as described above (*n* = 6 per group). **C.** The experimental mice in Figure 6B were sacrificed and tumor weight and metastatic nodules on lung tissues were determined at the 6^th^ week. **D.** mcCDRMB231 (MBR) cells were subcutaneously inoculated into nude mice. One week later, CDDP with or without RI37 or KT44 was administered to the experimental mice as indicated. Tumor size was measured as described above (*n* = 3 per group). **E.** The experimental mice in Figure 6D were sacrificed and tumor weight and metastatic nodules on lung tissues were quantified at the 16^th^ week. **F.** A schematic illustration of the paracrine crosstalk between stromal cells and cancer cells. Anticancer drugs induce PTX3 expression in stromal cells via activating CEBPD, leading to subsequent tumorigenicity properties including tumor metastasis, invasiveness, stemness and drug resistance.

The loss-of-function assay indicated that PTX3 contributed to CDDP- and 5FU-induced protumor effects in drug-susceptible and resistant breast cancer cells, respectively. In addition, a recent study showed that glycosylation of Asn220 is important for PTX3 regulation of inflammation and immunity [23, 32]. Recombinant PTX3 proteins purified from insect cells (bvPTX3) has no effect on the proliferation of MB231 cells (Figure. 5A and 5B), promotion of sphere-forming ability *in vitro* (Figure. 5C), enhancement of anticancer-drug resistance (Figure. 5D) and migration and invasion of mcCDRMB231 cells *in vitro* (Figure. 5E). Importantly, the recombinant bvPTX3 mutant of Asn220 (mbvPTX3) dramatically inhibited all the above bvPTX3-induced phenomena (Figure. 5C-5E). These results implied that glycosylation of PTX3 at Asn220 is critical for its protumor function. We next synthesized the PTX3 peptides RI37 (amino acids 200-236) and KT44 (amino acids 255-298) and tested their antitumor effects (Figure. 6A). We found that RI37 suppressed the growth, metastasis and invasion of drug-resistant cancers *in vivo* (Figure. 6B-6E). This study implied that RI37 may be an antagonist of PTX3-enhanced sphere-forming ability, anticancer-drug resistance, migration and invasion of cancer cells.

## DISCUSSION

Tumor shrinkage is usually accomplished with cancer therapy, but this benefit is transient and most patients eventually develop chemotherapy-resistant and widely disseminated cancer. As described above, CEBPD is induced by many inflammatory factors and in turn contributes to the activation of many inflammatory factors in macrophages [15, 33]. An increase in mammary tumor multiplicity and a decrease in lung metastasis were observed in *HER2/neu*Tg mice lacking CEBPD [19]. In the current study, we demonstrated a novel function of CEBPD: the molecule plays a protumor role in noncancerous cell types, such as fibroblasts and macrophages, in the context of the cancer microenvironment. Previously, CEBPD was suggested to be involved in M1 macrophage polarization [34]. Our preliminary data showed that the loss of CEBPD attenuated the differentiation of both M1 and M2 macrophages by verifying their macrophage markers following differentiation by GM-CSF or M-CSF-treated primary mouse monocytes (Ju-Ming Wang's unpublished results). The result indicated that CEBPD contributes to both M1 and M2 macrophage differentiation but the detailed regulation of CEBPD-mediated M1 and M2 macrophages needs to be further investigated. We showed that the activation of CEBPD in M2 macrophages and myofibroblasts/CAFs promoted sphere-forming ability and the metastasis and invasion of both responsive and drug-resistant cancers. Our data suggest that CEBPD plays an important role in microenvironment-mediated cancer progression. Our study also indicates that the dissection of CEBPD biology in the tumor microenvironment could suggest targets for developing anticancer reagents, such as the RI37 peptides demonstrated here, that may be used for cancer therapy in the future (Figure. 6F).

CAFs and TAMs increase tumor cell growth and motility, ultimately facilitating cancer cell escape from the primary tumor and thereby inducing metastatic spread. Recently, chemotherapies directed against some of these targets, which are overexpressed by breast tumor cells as well as by TAMs and CAFs in a tumor microenvironment, resulted in the elimination of tumor growth, progression, metastasis and recurrence in mouse tumor models. Previously, we suggested that PGE2-activated CEBPD may play a protumor role in the tumor microenvironment [17]. In this study, we found that CEBPD was also activated by CDDP and 5-FU in TAM and CAF cells (Figure. 1A). In addition to its functions in immunosuppression and inhibition of cancer cell phagocytosis by activated macrophages [17], we revealed a novel CEBPD function in the tumor microenvironment, namely, the promotion of sphere-forming ability, metastasis and invasion of drug-resistant cancers in CDDP- or 5-FU-treated TAMs and CAFs.

In response to extracellular stresses and inflammatory cytokines, several processes, including induction of cell migration, angiogenesis, immunosuppression and inhibition of phagocytosis, have been observed and are associated with inflammation. These inflammatory responses have also been linked to the initiation of cancer and cancer progression and even the occurrence of chemoresistance. However, the details of this link largely remain unclear, especially the responses associated with anticancer drug-induced inflammation-like environments. Pentraxin family members are characterized by a cyclic multimeric structure and contain a conserved carboxy-terminal domain called a pentraxin domain. In addition to its wide use as a marker of inflammation and infection, PTX3 has been observed at increased levels in several cancers, as mentioned previously. However, unlike the well-studied involvement of other pentraxin members in cancer [35, 36], the biology of PTX3 in cancer is less well understood.

Interestingly, as well as bvPTX3, our preliminary results showed that the recombinant PTX3 protein purified from mammal cells (euPTX3) has no effect on the proliferation of various cancer cells, but in agreement with the above observations, euPTX3 promoted sphere-forming ability *in vitro*, attenuated *E-cadherin* and increased *N-cadherin*, *Twist1* and *Snail2* transcripts, increased drug-resistance and migration and invasion of mcCDRMB231 cells *in vitro*. In addition, CD44 is also expressed in various types of cancer cells and cancer stem cells. Early observations have demonstrated a significant correlation between CD44 and tumor recurrence and mortality. CD44 may be an indicator of tumors and metastasis in malignant cancers [37, 38]. It has been speculated that PTX3 could form a complex with TSG6 and CD44 [39]. However, the involvement of CD44 in PTX3-induced tumorigenesis remains further investigation. As shown in Fig. S6, as well as mbvPTX3, RI37 has no direct effect on the growth of cancer cells. However, RI37 could attenuate the growth of xenografted cancers (Figure 6B and 6C). The *in vivo* result implies that PTX3 can play an autocrine/paracrine role to stimulate pro-growth factor(s) from stromal cells surrounding cancer cells.

In the current study, we further revealed involvement of PTX3 in the tumor microenvironment in promoting cancer metastasis, invasion and stemness. Our findings not only describe the biological significance of PTX3 in tumorigenesis but also suggest that PTX3 may be a key pro-tumorigenic player in the tumor microenvironment. In addition, the mutant bvPTX3 and RI37 further supported this speculation. Recently, a group showed that PTX3 inhibited FGF/FGFR-driven EMT in human A375 and A2058 melanoma cells [40]. They further demonstrated that PTX3 was a potent FGF antagonist and was endowed with anti-angiogensis and anti-neoplastic activity in mouse prostate TRAMP-C2 cancer cells [41]. As well as MB231, A549 and HONE1 cells, our results also showed that euPTX3 promoted the migration and invasion of prostate cancer PC3 and LNCaP cells (Figure. S7A and S7B). Several recent studies mentioned above [25-27] and the current study supported the protumor role of PTX3, we still cannot rule out the possibility that the protumor role of PTX3 may act in a cell line-, tumor- or species-specific manner.

Patients with recurrent cancers, especially those with anticancer drug-resistant cancers, are always at a higher risk because these cancers always spread faster than the original tumor. Nevertheless, the mechanisms related to this issue are less understood and largely uninvestigated. The activation of CEBPD induces apoptosis of cancer cells [18, 42]. Comparing to the activation of CEBPD in tumor-associated stromal cells, the activation of CEBPD is insensitive in breast cancer cells because an epigenetic-mediated hypermethylation on the promoter region of *CEBPD* gene [43, 44]. The hypermethylated *CEBPD* promoter can attenuate the responses of CBEPD and its downstream targets in cancer cells [18]. Insufficient activation of CEBPD in cancer cells has been suggested to induce genomic instability [14]. The current study further supports our previous study [17] showing CEBPD serves a protumor role in tumor microenvironment. Therefore, the dual role of CEBPD in both killing and promoting cancer has led us to think more about how to apply and optimize “CEBPD reactivation” to provide a useful and safe solution when administering these chemotherapeutic drugs. The CEBPD downstream target PTX3 that we focused on here can provide a great opportunity for developing a feasible translational application for cancer therapy.

## MATERIALS AND METHODS

### Cell culture and treatment

THP-1 cells were cultured in RPMI 1640 medium (Hyclone). Human lung fibroblasts (HFL1) were obtained from the American Type Culture Collection (ATCC). HFL1 cells were cultured in Ham's F-12 Kaighn's Modification medium (Gibco). The CAF/F28 was established from a patient [45]. Mouse embryo fibroblasts (MEFs) used in this study were isolated from individual E13.5-E14.5 embryos generated by mating *Cebpd* null heterozygous mice and immortalized with E1A [46]. The CAF/F28 cells, human breast cancer MB231 cells, mouse breast cancer 4T1 cells and immortalized *Cebpd*^+/+^ (7V7) and *Cebpd*^−/−^ (KO5) MEFs were maintained in DMEM. All culture media were supplemented with 10% FBS, streptomycin and penicillin. To generate THP-1/M2 macrophages, THP-1 cells were treated with 320 nM PMA for 6 h and then cultured with PMA plus 20 ng/ml IL-4 and 20 ng/ml IL-13 for another 18 h [47]. To generate primary mouse M2 macrophages, mononuclear cells obtained from bone marrow were treated with 25 ng/ml M-CSF in RPMI 1640 medium with 10% FBS [48]. To generate myofibroblasts, HFL1 cells or MEFs were treated with 5 μg/ml TGF-β for 3 days. The following concentrations of peptide were used in this study: 10 μg/ml PTX3 peptide (RI37 or KT44). The anticancer drugs were then added individually for each experiment: 40 μM CDDP or 100 μM 5-FU.

### Conditioned medium collection

For the loss-of function assay, conditioned medium was collected from THP-1/M2 or CAF/F28 cells infected with lentiviruses bearing shLacZ, shCEBPD or shPTX3 after 48 h. The experimental cells were further treated with or without CDDP or 5-FU for 24 h, which was replaced with serum-free RPMI medium (conditioned medium from THP-1 cells and mouse primary macrophages), 0.5% serum in F12K medium (conditioned medium from HFL1 cells) or 0.5% serum in DMEM medium (conditioned medium from CAF/F28 cells) for another 24 h. Following centrifugation, the supernatants were collected and stocked for further assays in this study. For the gain-of-function assay, conditioned medium was collected from THP-1/M2 (M2MΦ) or CAF/F28 cells 24 h after infection with lentiviruses bearing an empty vector or CEBPD expression vector. Then, the cells were prepared as described above.

### Reporter plasmids and luciferase assay

The *PTX3* reporter was constructed by cutting the DNA fragment of the *PTX3* promoter from pGL2-basic/PTX3 [49] with KpnI and HindIII and subcloning the fragment into the pGL3-basic vector. For the reporter assay, cells were transiently transfected using the *PTX3* reporter and indicated expression vectors with the TransIT-2020 transfection reagent (Mirus) according to the manufacturer's instructions. Eighteen hours following transfection, the transfectants were treated with CDDP or 5-FU for another 6 h. The lysates of experimental cells were harvested to conduct the luciferase assay.

### ChIP assay

The ChIP assay was performed essentially as described by Wang et al. [50]. Briefly, following various treatments, the experimental THP-1/M2 or CAF/F28 cells were fixed with 1% formaldehyde. The cross-linked chromatin was then prepared and sonicated to an average size of 500 bp. The DNA fragments were immunoprecipitated with antibodies specific for CEBPD or control rabbit immunoglobulin G at 4 °C overnight. After reversal of the cross-links, the immunoprecipitated chromatin was amplified by primers targeting specific regions of the gene's genomic locus. The primers used detected sequences in the *PTX3* genomic locus (5′-GCTCGGATTGGACTTGACTT-3′ and 5′-GAGGGAAATGTGGAAGTTGC-3′). The amplified DNA products were resolved by agarose gel electrophoresis and confirmed by sequencing.

### Purification of recombinant PTX3 mutant proteins

The same primers were used for cloning and for expression in *E. coli*. We cloned partial full-length (19-381) coding regions of the PTX3 gene into the pFastBac vector. To generate mutant constructs by site-directed mutagenesis, we used the following primers: forward primer 5′-GCTAAAACCATCCTGTTTTCCTATG-3′ and reverse primer 5′-TAATACATCTGTGGCTTTGACCCAA-3′. Various baculoviruses containing recombinants of PTX3, including the mutant form (N220A), were produced in DH10BAC cells and then were amplified and used to infect Sf9 insect cells in serum-free media (Invitrogen). The cells were cultured in suspension and harvested 40 h after infection. The recombinant proteins were purified by nickel affinity chromatography and gel filtration chromatography.

### Cell viability assay

To assess the viability of cancer stem cells in response to anticancer drugs using the MTT [3-(4,5-dimethylthiazol-2)-2,5-diphenyltetrazolium bromide] assay (Sigma), cancer cells were pretreated with various conditioned media as described above with PTX3 peptide as indicated in each experiment for 7 days. Then, 1×10^4^ experimental cells were treated with CDDP or 5-FU for another 48 h. To assess the proliferation of cancer cells using the MTT assay, cancer cells were treated with PTX3 peptides as indicated in each experiment for 24 and 48 h.

### Migration assays and matrigel invasion assay

For the migration assay, cells were seeded (3 × 10^4^ for mcMB231, mcCDRMB231 and mcFURMB231 cells or 5 × 10^4^ for mCherry fluorescent 4T1, mcCDR4T1 and mcFUR4T1 cells) in 24-well plates containing 8-μm pore inserts (BD Biosciences). For the matrigel invasion assay, the 8-μm pore inserts of the 24-well plates were pre-coated with appropriate matrigel and the same cell number as mentioned in the migration assay were seeded onto the matrigel. Serum-free conditioned medium was placed in the upper wells after cells adhered to the inserts. DMEM with 10% FBS was added to the lower wells of the 24-well plates. The cells inside the insert were wiped with cotton swabs and removed after 16 hours of incubation. The cells that had migrated to the bottom of the insert membrane were detected by DAPI. The total number of cells attached to the lower surface of the polycarbonate filter insert was counted at 200X magnification under a fluorescence microscope.

### Reverse-transcriptase polymerase chain reaction (RT-PCR)

Total RNA of experimental cells was isolated using TRIzol RNA extraction reagent. For RT-PCR analysis, total RNA was subjected to reverse transcription with SuperScript III (Invitrogen). The specific oligonucleotide primers used for the RT-PCR analysis are as follows: CEBPD, 5′-GCCATGTACGACGACGAGAG-3′ and 5′-TGTGATTGCTGTTGAAGAGGTC-3′; GAPDH, 5′-CCATCACCATCTTCCAGGAG-3′ and 5′-CCTGCTTCACCACCTTCTTG-3′; and PTX3, 5′-TGTTGAGATGGCCACAGG-3′ and 5′-TCAGACCTTCCCAACTGG-3′.

### Western blotting analysis

Cells were lysed in modified radioimmunoprecipitation assay buffer (modified RIPA) [50 mM Tris-HCl (pH 7.4), 150 mM NaCl, 1 mM EDTA, 1% NP-40, 0.25% sodium deoxycholate, 1 mM dithiothreitol, 1 mM phenylmethylsulfonyl fluoride, aprotinin (1 mg/ml) and leupeptin (1 mg/ml)]. Specific antibodies including α-tubulin (T6199, Sigma) and CEBPD (sc-636, Santa Cruz Biotechnology) were used for Western blotting.

### Short hairpin RNA (shRNA) assay

Lentiviruses were produced from Phoenix cells that had been cotransfected with various shRNA expression vectors in combination with pMD2.G and psPAX2. After determining the viral infection efficiency, THP-1/M2 and CAF/F28 cells were infected for 48 h with shC, shD or shP lentiviruses, each at a multiplicity of infection (MOI) of 10. The shRNA oligo sequences used in the lentiviral expression vectors were as follows: shC, 5′-CCGGTGTTCGCATTATCCGAACCATCTC GAGATGGTTCGGATAATGCGAACATTTTTG-3′; shD, 5′-CCGGGCTGTCGGCTGAGAACGAGAACT CGAGTTCTCGTTCTCAGCCGACAGCTTTTT-3′; and shP, 5′- CCGGGAGGAGCTCAGTATGTTTCATCTCGA GATGAAACATACTGAGCTCCTCTTTTTTG-3′. The lentiviral knockdown expression vectors were purchased from the National RNAi Core Facility located at the Genomic Research Center of the Institute of Molecular Biology, Academia Sinica, Taiwan.

### Sphere formation assay

Cancer cells were cultured with conditioned medium as described above. Then, cells were plated in ultralow attachment plates (Corning) at a density of 5×10^3^ cells per well. Cells were grown in serum-free DMEM/F12 (Gibco) supplemented with B27 (Invitrogen), 20 ng/mL EGF (Abcam) and 10 ng/mL bFGF (Peprotech). After two weeks, the total number of spheres was counted at 100X magnification under a microscope.

### Xenograft tumor formation from spheres in NOD-SCID mice

Six-week-old female NOD-SCID mice were subcutaneously injected with spheres of breast cancer cells at low doses of 10^4^ or 10^3^ viable cells. Animals were sacrificed 1 month following inoculation. All experiments on mice were performed according to the guidelines of our institute (Guide for Care and Use of Laboratory Animals, National Cheng Kung University). The animal use protocol described has been reviewed and approved by the Institutional Animal Care and Use Committee (IACUC).

### Xenograft animal study

In mcCDRMB231 xenografts, 2 × 10^6^ mcCDRMB231 cells mixed with 5 × 10^5^ shC-M2 macrophages, 5 × 10^5^ shD-M2 macrophages, 5 × 10^5^ shC-CAFs or 5 × 10^5^ shD-CAFs were inoculated subcutaneously into the dorsal rear flanks of NOD-SCID mice. In mcCDR4T1 xenografts, 1 × 10^6^ mcCDR4T1 cells were mixed with 5 × 10^5^ 7V7 MEF-derived myofibroblasts, 5 × 10^5^ KO5 MEF–derived myofibroblasts, 5 × 10^5^ *Cebpd*^+/+^ bone marrow–derived M2 macrophages or 5 × 10^5^ *Cebpd*^−/−^ bone marrow–derived M2 macrophages and inoculated subcutaneously into the dorsal rear flanks of NOD-SCID mice. In *in vivo* experiments of mice treated with or without CDDP, beginning 1 weeks after inoculation with tumor cells, mice were treated weekly with intraperitoneal injection of CDDP (5 mg/kg) dissolved in 1% (w/v) DMSO or DMSO only. Tumor size was measured with external calipers and tumor volume was calculated using the standard formula: V = (w × l^2^) × 0.52, where l is the length and w is the width of the tumor. The animals were sacrificed after tumors cells had been inoculated for 5-12 weeks. The number of the metastasized tumor nodules on lung surface was quantified. All experiments on mice were performed according to the guidelines of our institute (the Guide for Care and Use of Laboratory Animals, National Cheng Kung University). The animal use protocol listed below has been reviewed and approved by the Institutional Animal Care and Use Committee (IACUC).

### Allograft animal study

6- to 8-week-old female BALB/c mice were used in this assay. One million mcCDR4T1 cells were inoculated subcutaneously into the dorsal rear flanks of BALB/c mice. In *in vivo* experiments of mice treated with or without CDDP, beginning 1 weeks after inoculation with tumor cells, mice were injected intraperitoneally every week with CDDP (5 mg/kg) dissolved in 1% (w/v) DMSO or DMSO only. The tumors were then subcutaneously injected with PTX3 peptides RI37 (50 μg) or KT44 (50 μg) 3 times per week. Tumor size was measured with external calipers and tumor volume was calculated using the standard formula: V = (w × l^2^) × 0.52, where l is the length and w is the width of the tumor. Animals were sacrificed after tumor cells had been inoculated for 6 weeks. The number of the metastasized tumor nodules on lung surface was quantified. All experiments on mice were performed according to the guidelines of our institute (the Guide for Care and Use of Laboratory Animals, National Cheng Kung University). The animal use protocol listed below has been reviewed and approved by the Institutional Animal Care and Use Committee (IACUC).

## SUPPLEMENTARY MATERIAL FIGURES


