# Supplementary material for: Targeting chemotherapy-induced PTX3 in tumor stroma to prevent the progression of drug-resistant cancers
Source: Oncotarget. 2015 Jun 8;6(27):23987–4001. doi: 10.18632/oncotarget.4364 (PMC4695165; doi:10.18632/oncotarget.4364)
Supplement: Supplementary file 1 [file oncotarget-06-23987-s001.pdf]

Targeting chemotherapy-induced PTX3 in tumor stroma to prevent the progression of drug-resistant cancers

Supplementary Material

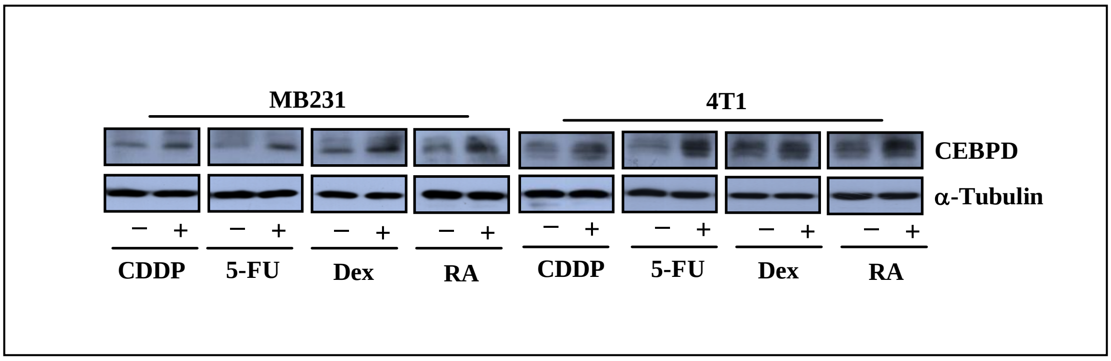

Figure S1: Anticancer drugs induce CEBPD expression in breast cancer cells. MDA-MB231 (MB231) and 4T1 cells were treated with CDDP (30 μM), 5-FU (10 μg/ml), dexamethasone (Dex; 1 μM) or retinoic acid (RA; 10 μM) for 6 h. Total lysates were harvested for Western blot analysis using CEBPD and α-tubulin antibodies.

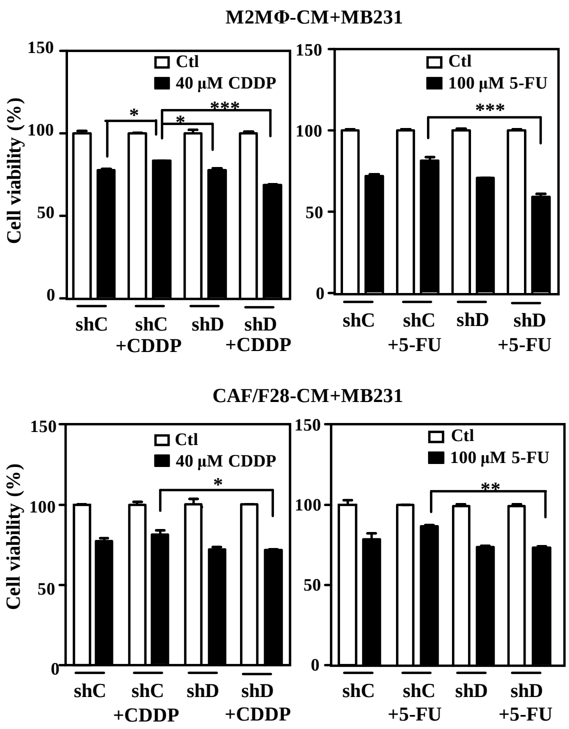

Figure S2: CEBPD-expressing M$_2$-like macrophages or CAFs promote drug resistance of breast cancer cells after anticancer drug treatment. The conditioned medium from THP-1/M2 or CAF/F28 cells lacking CEBPD significantly decreased the viability of MB231 after treatment with CDDP or 5-FU. The viability of MB231 cells was increased in the treatment with CDDP or 5-FU in the context of conditioned medium from THP-1/M2 or CAF/F28 cells infected with shC or shD lentiviruses with or without CDDP or 5-FU.

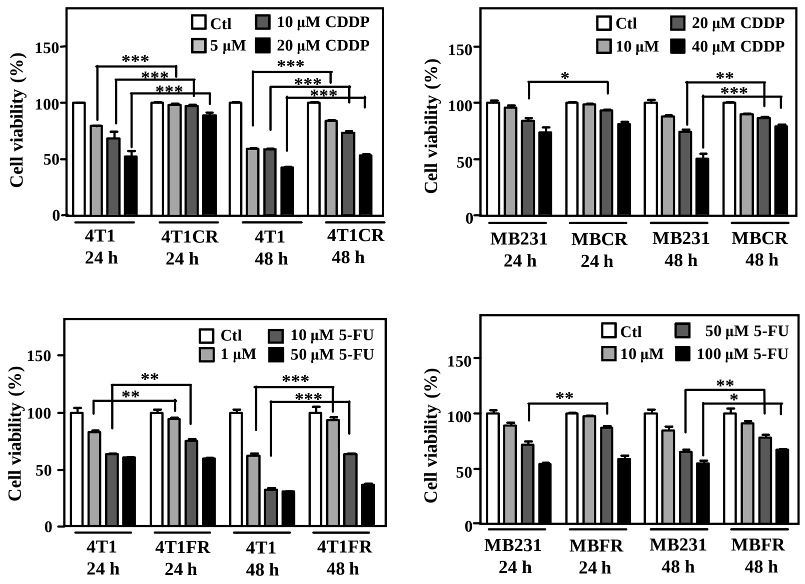

Figure S3: The viability of parental mCherry fluorescent 4T1 (4T1) and mcMB231 (MB231) cells and drug-resistant mcCDR4T1 (4T1CR), mcFUR4T1 (4T1FR), mcCDRMB231 (MBCR) or mcFURMB231 (MBFR) cells was measured using a cell viability assay.

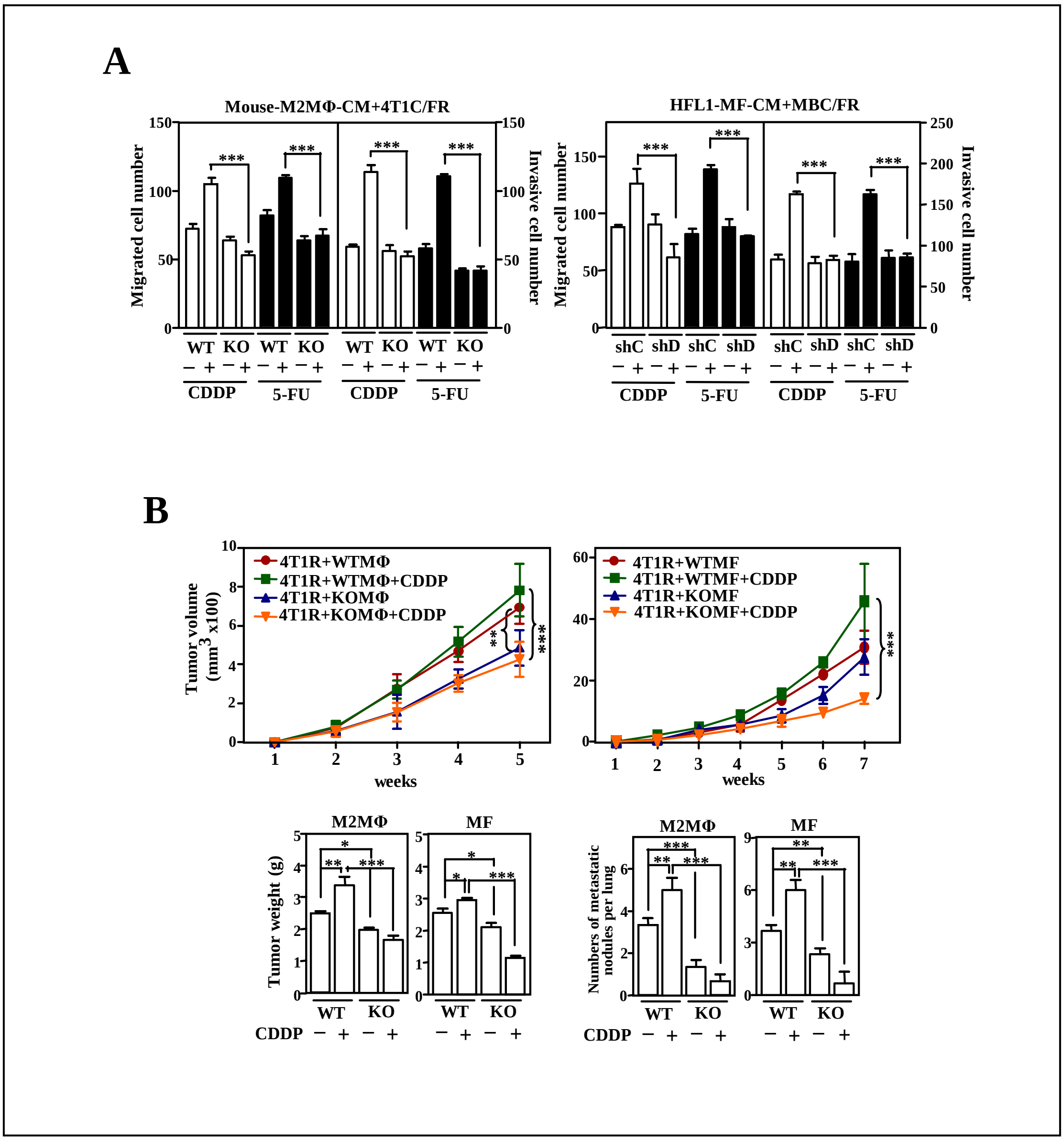

Figure S4: CEBPD-expressing M$_2$ macrophages or myofibroblasts promote cancer growth and metastasis/invasion after anticancer drug treatment. (A) The migration and invasion of drug-resistant cancer cells were assessed using a Boyden chamber assay in which the cells were cultured in conditioned medium from M-CSF-differentiated *Cebpd*$^{+/+}$ or *Cebpd*$^{-/-}$ M2 macrophages (mouse M2MΦ) or TGF-β-differentiated HFL myofibroblasts (HFL-MF) infected with shC or shD lentiviruses with or without CDDP or 5-FU treatment. **(B)** M-CSF-differentiated *Cebpd*$^{+/+}$ or *Cebpd*$^{-/-}$ M2 macrophages or TGF-β-differentiated immortalized 7V7 or KO5 myofibroblasts were subcutaneously co-inoculated with mcCDR4T1 (4T1R)

cells into NOD-SCID mice that were treated with or without CDDP (three times per week). Tumor size and metastatic tumors were assessed as described above (n=6 in per group).

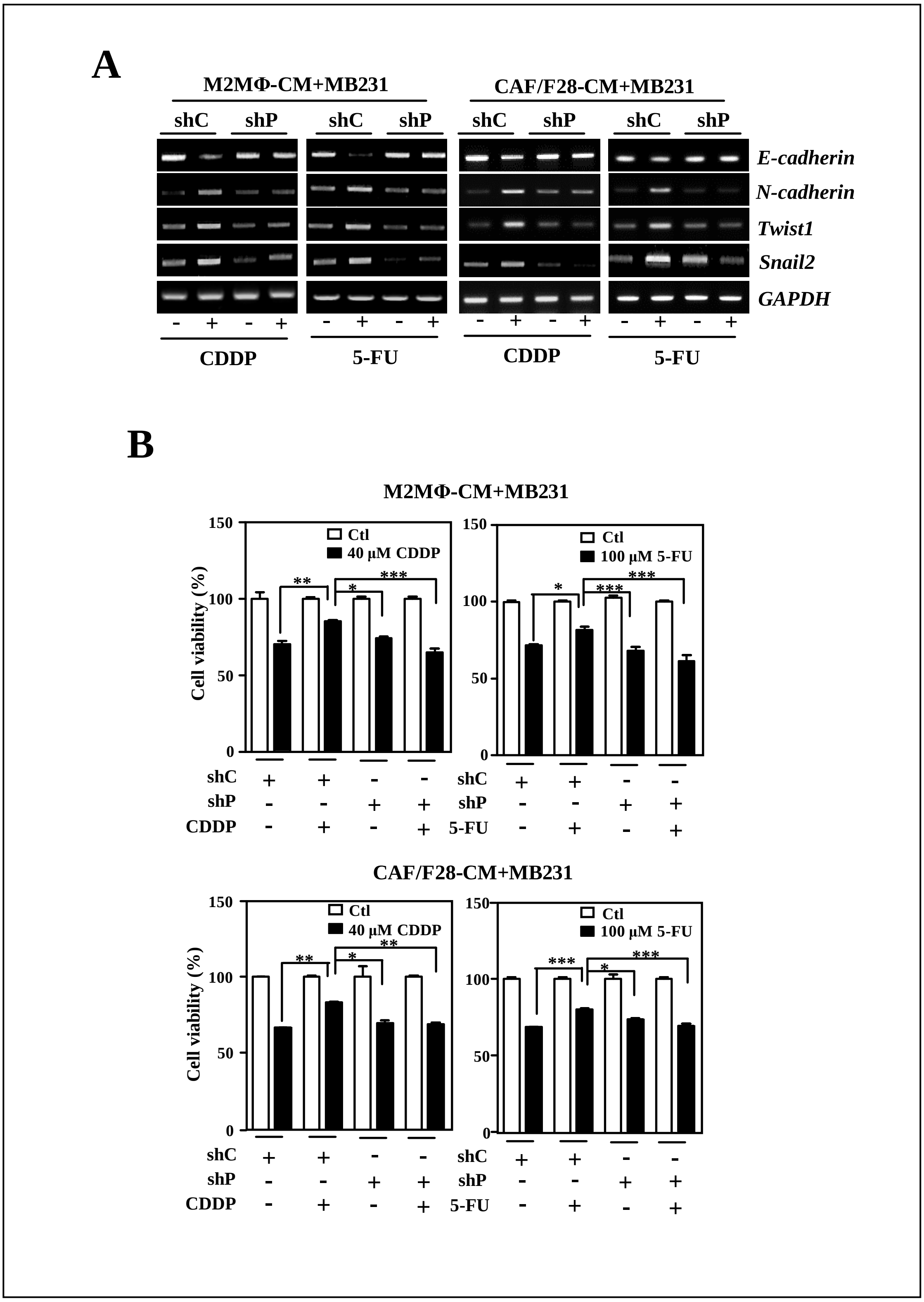

Figure S5: PTX3-expressing M2-like macrophages or CAFs promote the EMT and drug resistance of breast cancer cells upon anticancer drug treatment. (A) PTX3-expressing THP-1/M2 or CAF/F28 cells regulated transcription of EMT (*E-cadherin* and *N-cadherin*) and cancer stem cell (*Twist1* and *Snail2*) markers in

MB231 cells. An RT-PCR assay was conducted on total RNA harvested from MB231 cells cultured with conditioned medium from THP-1/M2 or CAF/F28 cells infected with shC or shP lentiviruses and treated with or without CDDP or 5-FU. **(B)** The conditioned medium from THP-1/M2 or CAF/F28 cells lacking PTX3 significantly decreased the viability of MB231 after treatment with CDDP or 5-FU. The effect of viability of MB231 cells after treatment with CDDP or 5-FU in the context of conditioned medium from THP-1/M2 or CAF/F28 cells infected with shC or shP lentiviruses with or without CDDP or 5-FU.

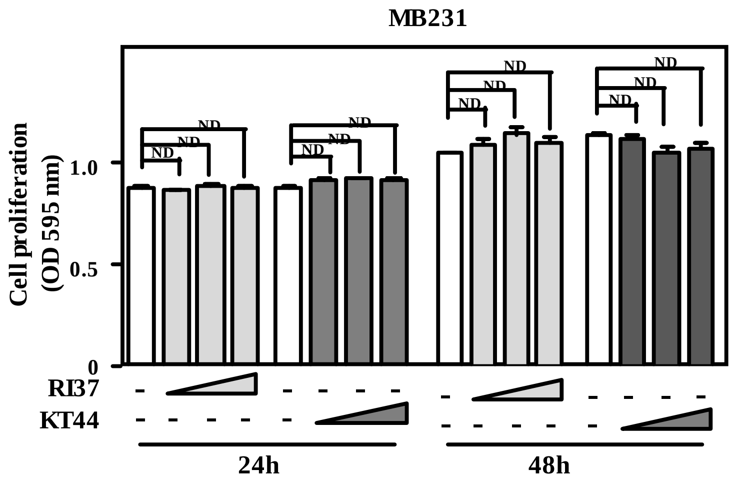

Figure S6: PTX3 peptides have no effect on the growth of cancer cells. The effects of the indicated peptides were tested in MB231 cells. The cell proliferation was conducted after the indicated cells were treated with RI37 or KT44 at 24th h and 48th h.

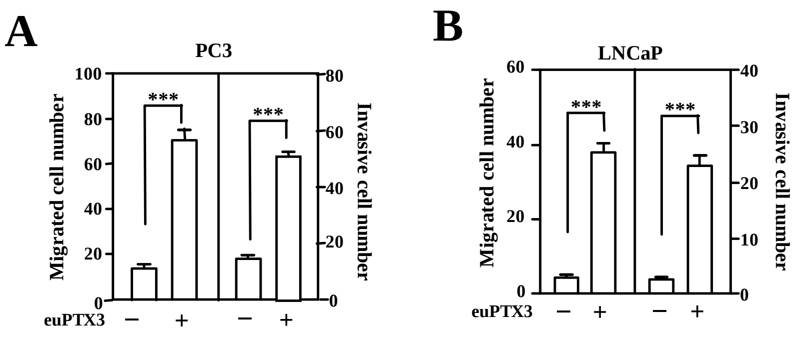

Figure S7: Effects of euPTX3 on the migration and invasion of prostate cancer cells. (A) PC3 and **(B)** LNCaP cells were seeded in the upper layer of a Boyden chamber. After 3 h, the culture medium was replaced with serum-free medium in the upper layer and the indicated amount of euPTX3 was added to serum-free medium in the bottom layer. After 16 h of incubation, the cells that migrated to the bottom layer were stained with DAPI and counted using a fluorescence microscope.
